# COVID-19 mortality in patients with immunodeficiency and its predictors: a systematic review

**DOI:** 10.1186/s40001-022-00824-7

**Published:** 2022-10-08

**Authors:** SeyedAhmad SeyedAlinaghi, Amirali Karimi, Alireza Barzegary, Hengameh Mojdeganlou, Farzin Vahedi, Seyed Peyman Mirghaderi, Parnian Shobeiri, Maryam Ramezani, Parisa Yousefi Konjdar, Pegah Mirzapour, Marcarious M. Tantuoyir, Esmaeil Mehraeen, Omid Dadras, Fabricio Voltarelli

**Affiliations:** 1 Iranian Research Center for HIV/AIDS, Iranian Institute for Reduction of High Risk Behaviors, Tehran University of Medical Sciences, Tehran, Iran; 2 School of Medicine, Tehran University of Medical Sciences, Tehran, Iran; 3 School of Medicine, Islamic Azad University, Tehran, Iran; 4 Department of Pathology, Urmia University of Medical Sciences, Urmia, Iran; 5 Department of Health Management, Policy & Economics,, School of Public Health, Tehran University of Medical Sciences, Tehran, Iran; 6 Department of Health Information Management, Faculty of Paramedical, Kashan University of Medical Sciences, Kashan, Iran; 7 Biomedical Engineering Unit, University of Ghana Medical Center (UGMC), Accra, Ghana; 8 Department of Health Information Technology, Khalkhal University of Medical Sciences, Khalkhal, Iran; 9 Department of Global Public Health and Primary Care, University of Bergen, Bergen, Norway; 10 Graduation Program in Health Sciences, Faculty of Medicine, Federal University of Mato Grosso, Cuiabá, Mato Grosso Brazil

**Keywords:** COVID-19, Immunocompromised, Immunodeficiency, Immunosuppression, Impaired immune response, SARS-CoV-2, Comorbidities

## Abstract

**Introduction:**

Patients with immunodeficiency are usually more prone to worse outcomes of infectious diseases. However, there are some disagreements in the context of COVID-19, for example, in patients with human immunodeficiency virus (HIV). Herein, we aimed to systematically review the risk and predictors of COVID-19 mortality in people with primary or secondary immunodeficiency.

**Methods:**

PubMed, Scopus, Web of Science, and Science Direct were searched. We followed a two-step screening process to identify eligible results. We first reviewed the title and abstract of the records and the unqualified studies were removed. Then, their full texts were evaluated based on their coherence with the purpose and inclusion/exclusion criteria, and those eligible for qualitative synthesis were included.

**Results:**

Twenty-two articles were included, which investigated a total of 109,326 with primary or secondary immunodeficiencies. Three studies investigated the pediatric and infant population, while other studies were conducted on the adult population. Overall, studies on both primary and secondary immunodeficiency conflicted as some reported higher and some mentioned lower mortality rates in patients with immunodeficiency.

**Conclusions:**

Overall, there were two points of view in both types of immunodeficiencies. The first is the classical viewpoint that all immunodeficient patients are at a higher risk of infection leading to a higher mortality rate. The second types of studies found that immunodeficiency might play a less important or even an inverse role in mortality rates by lowering the severity of the inflammatory response. However, it is important to take note to comorbidities, such as DM, HTN, CAD, ESRD, history of lower respiratory infection, etc., and demographic factors, such as obesity and age > 70 years, as they appear to influence the mortality rate, especially in patients with secondary immunodeficiency.

## Introduction

Since December 2019, a fatal respiratory disease named coronavirus 2019 (COVID-19) has spread worldwide [1, 2]. It has quickly progressed from a few pneumonia-like cases to a pandemic that is still spreading. Humoral immune response, in particular the creation of neutralizing antibodies, serves as a protective mechanism by delaying the onset of infection [3–5]. Severe acute respiratory syndrome-Coronavirus 2 (SARS-CoV-2) infection can potentially influence a wide range of human organs, and infected people may show a sort of symptoms that may vary from person to person [6–9]. Apart from the tremendous potential of the novel coronavirus in impairing the respiratory system, its quick propagation and propensity to engage numerous hosts in severe cases of infections or immunopathological consequences make it a challenging opponent for the immune system [10]. The interplay between SARS-CoV-2 and the host immune system result in unleashing massive numbers of cytokines secretion [11, 12]. The cytokine storm introduces a dangerous situation that determines the clinical course (healing or mortality) and may lead to multi-organ failure [13, 14].

Some studies claimed that immunodeficient and immunocompromised patients have poorer prognoses and outcomes and higher mortality rates than healthy people, but the outcome and prognosis mainly depend on the type and level of immunodeficiency. Moreover, the wide range of microorganisms that cause infection has different prognoses in immunodeficient people [15, 16]. Regarding COVID-19, however, conflicting results exist between immunodeficiency and the risk of severe outcomes [17–19].

Considering the current COVID-19 pandemic, it is highly important to improve the surveillance and management of immunodeficient people to reduce mortality and morbidities among them [19]. Therefore, actions must be developed based on scientific facts to decreased devastating outcomes, and this requires a comprehensive review of current literature to provide precise prediction models and improve the outcomes of COVID-19 infection in immunocompromised patients. As clinicians, we should assess the patterns of mortality and comorbidities in immunodeficient patients to design a well-organized system to provide care when needed [20]. This assessment of patterns of the disease might be the key to reduce its mortality and comorbidities. For instance, the United Kingdom Primary Immunodeficiency Network has developed a system to systematically follow PID (primary immunodeficiency) and SID (secondary immunodeficiency) patients and record their COVID-19 infection status as well as outcomes [21]. Given this background, we systematically reviewed studies on immunodeficient people infected with COVID-19 regarding their risk factors and subsequent predictors of mortality rates**.**

## Materials and methods

This study is a systematic review of current literature conducted in March 2021. The authors studied COVID-19 mortality in patients with immunodeficiency and its predictors. Our study was conducted according to the Preferred Systematic Review and Meta-Analysis Report (PRISMA) to ensure the reliability and validity of the results.

### Data sources

Using systematic searches and keywords in online databases, including PubMed, Scopus, Web of Science, and Science Direct, we extracted all relevant articles published in English up to March 2021. We have included several combinations of keywords in the following commands to perform a search strategy: (1) “COVID-19” or “SARS-CoV-2” [title/abstract]; (2) “Immunocompromised” [Title/Abstract] (3) “Immunodeficiency” [Title/Abstract] (4) “Immunosuppression” [Title/Abstract] (5) “Impaired immune response” [Title/Abstract].

### Inclusion/exclusion criteria

The original, English, and peer-reviewed studies that reported the outcomes of COVID-19 in patients with immunodeficiency were included.

Exclusion criteria were as follows: (1) abstracts or the articles whose full-text was not available, (2) text written in any other language except English, (3) and non-original articles (editorials, reviews, etc.), (4) studies that were not peer-reviewed, or (5) animal or laboratory (non-human) studies.

### Study selection

Two independent researchers selected relevant studies screening the titles and abstracts of the retrieved records. After that, the full-text of the retrieved articles was reviewed, and the most relevant articles were selected according to the eligibility criteria. Then, we extracted the relevant data and organized them into tables.

### Data extraction

After summarizing, we transferred the study information, the first author (reference), type of study, country, study population, age(average), gender, primary immunodeficiency, secondary immunodeficiency, laboratory factors, clinical factors, underlying disease, and mortality rate (*N*(%)) to a data sheet. Two independent researchers collected this information and subsequently organized it into tables. Finally, all articles selected by other authors were reviewed to ensure that there was no duplication or overlap in the content.

### Risk of bias/quality assessment

We assessed the risk of bias/quality of the studies using the Newcastle–Ottawa scale (NOS) checklist. This scale allocates a 0–9 score to each study based on selection, comparability, and exposure/outcome [22]. Studies that scored 4 or below were considered of poor quality.

## Results

**Fig. 1:**
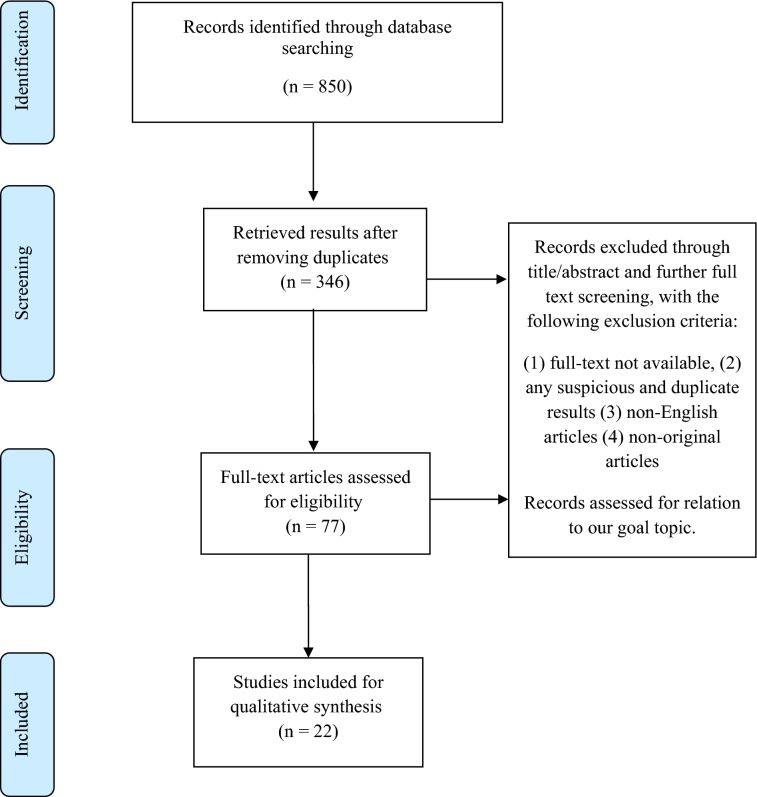
Prisma flow diagram of the study’s selection process

A total of 850 articles were retrieved from our comprehensive search strategy. After removing duplicates and screening the title/abstract followed by full-text evaluation for eligibility, 22 articles were included in the final narrative data synthesis. The exact details of the selection process are illustrated in Fig. 1.

The study design distribution of the selected studies is represented in Table 1, which were mainly retrospective studies. Most of the studies were conducted in the USA (*n* = 10), UK (*n* = 4), and Spain (*n* = 2). The selected studies investigated a total of 109,326 patients cumulatively, that suffered from primary immunodeficiency (common variable immune deficiency [CVID], humoral immunodeficiencies, etc.) or secondary immunodeficiency (HIV, malignancy, etc.). Three studies investigated the pediatric and infant populations [21, 23, 24], while other studies were conducted on the adult population. Table 2 displays the information extracted from the included articles.

**Table 1:** Study design distribution

Study design	Number (percent)
Retrospective cohort	9 (41%)
Cohort study	5 (23%)
Case-series	4 (18%)
Case-report	3 (14%)
Cross-sectional	1 (4%)

**Table 2:** COVID-19 mortality in patients with immunodeficiency and its predictors

ID	First author (reference)	Study population	COVID-19 mortality details								Mortality Rate [N (%)]
			Age(average)	Gender	Primary immunodeficiency	Secondary immunodeficiency	Laboratory factors	Clinical factors	Underlying disease	Other	
1	Bhaskaran K [17]	*N* = 17,282,905 [HIV-infected *N* = 27,480 (0·16%)]	Median = 48 (40–45)	Male (*n* = 17780 (64.7%)	–	HIV	–	Current or former Smoker (51.2%)	Hypertension (19.3%) Chronic respiratory disease (4.0%) Cardiovascular disease (3.4%) Stroke or dementia (2.0%) Other neurological disease (0.9%) Organ transplant (0.3%) Asplenia (0.3%) RA, lupus, or psoriasis (4.5%) Other immunosuppressive (0.2%) Diabetes mellitus (9.8%) non-hematological cancer (4.7%) Hematological malignancy (2%) chronic Renal failure (0.5%)	HIV-infected patients have higher risk of COVID-19 death: hazard ratio (HR) = 2·90 the association was larger in Black individuals (HR = 4·31)	0.09%
2	Cabello A [18]	*N* = 18853(HIV-infected = 63)	Median = 46 (37–56)	Male *n* = 56 (88.9%)	–	HIV	Lymphopenia (26.5%) Ferritin > 1000 mcg/L (25%) D-dimer > 2500 ng/mL (4.8%) CD4 + < 200 cells/µL (6.3%) Nadir CD4 < 200 cells/µL (25.4%)	HIV infection time (years) = 10.8 ART *N* = 61(96.8%) virological suppression time (years) = 7.2 Current or former Smoker (48.2%) COVID-19 diagnosis: laboratory confirmed (49.2%), clinically suspected (50.8%) duration of symptoms (days) before treatment = 6 Pneumonia (47.5%) WHO COVID-19 severity score (28.6%) Hospital admission (32.3%)	Previous comorbidity (84.1%) Hypertension (19%) Diabetes mellitus (9.5%) Overweight 13.1% cardiovascular disease (12.7%) Chronic respiratory disease (4.8%) chronic Renal failure (3.2%)	COVID-19 Prevalence in HIV-patients = 1.68% HIV-related factors did not show association with the severity of COVID-19	3.17%
3	Childs K [28]	*N* = 18	Median = 52(49–58)	Male *n* = 12 (67%)	–	HIV	Preadmission CD4 count, cells/µL = 395 Nadir CD4 count, cells/µL = 97 HIV RNA < 50 copies/ml = 17(94%) Lymphocytes = 1100 CRP = 143 (72–253)	HIV infection time (years) = 14.6 Smoker = 0 (0%) duration of COVID-19 symptoms at admission (day) = 8 Cough (72%) Dyspnea (67%) Fever (61%) Pneumonia (72%) AKI (28%) requiring mechanical ventilation = 5 (28%)	Obesity (BMI > 30 kg/m^2^) (56%) Hypertension (33%) Diabetes mellitus (22%) Chronic kidney disease (28%)	COVID-19 impose more morbidity and mortality on HIV-infected patients	27.8%
4	Cohen B[19]	*N* = 135 N = 10 (COVID-19 positive)	37	Male = 6 (60%)	CVID	–	Lymphopenia 3 (30%)	Pneumonia (0%) requiring mechanical ventilation = 0 (0%) mild to moderate symptoms = 9 (90%) on biological therapies = 2(20%)	Previous comorbidity (HTN, DM) = 3 (30.0%)	All the patients recovered CVID patients are not at a higher risk for mortality and worse outcome	0%
5	Del Amo J [32]	*N* = 77,590 (HIV-infected persons receiving ART) *N* = 236 (COVID-19 positive)	48.9	Male = 58,120 (75)	–	HIV	–	Hospital admission 151 (64%) median duration of hospitalization for discharged patients (day) = 7 (4–10) ICU admission 15 (6%), death: 20 (8%)	–	Standardized risk per 10,000 between the 77,590 HIV-positive persons = 30.0 for COVID-19 diagnosis = 3.7 for death greater risk in men and age > 70 years lower risk (= 16.9) for TDF/FTC regime incidence of COVID-19 in HIV infected population is comparable to the normal population TDF/FTC treatment may have beneficial impact for patients with COVID-19 and HIV co-infection	8%
6	Delavari S [24]	*N* = 2754 with primary immunodeficiencies (PIDs) *N* = 19 (0.68%, PIDs with COVID-19 positive)	9	Male = 1756 (63.8%)	Primary immunodeficiencies (PIDs): Combined immunodeficiencies (*n* = 1392) humoral immunodeficiencies (*n* = 1391) phagocytic defects (*n* = 782) immune dysregulation (*n* = 117) autoinflammatory disorders (*n* = 734) complement deficiency and innate immunodeficiencies (*n* = 304)	–	Negative acute-phase reactant proteins = 8 (42.1%)	Requiring respiratory support = 10 (52.6%) bronchiectasis 4 (21.0%) cardiovascular complications 2 (10.5%) liver failure 2 (10.5%) pulmonary complications (varied from mild prominence of broncho-vascular markings to mucus plugging, prebronchial thickening, diffuse patchy opacities, collapse/consolidations, mosaic perfusion, and ground glass interstitial disease, based on the severity of diagnosed PID)	history of lower respiratory tract infection before COVID-19 (89.4%) lymphoproliferation = 7 (36.8%)	1.23 folds higher risk of COVID-19 infections (0.68%) Tenfolds higher mortality rate (0.003%) most mortality rate of COVID-19 infection: SCID patient (0.03%) and FHL (0.027%)	42.1%
7	Fill L [25]	A CVID COVID-19 positive patient (confirmed by PCR)	53	Female	CVID	–	Leukopenia (2800 cells/µL) Lymphopenia (770 cells/µL) CBC, electrolytes, renal and liver function, and serum procalcitonin = in the normal range CRP = 16.6 mg/dL IgG = 1710 mg/dL (normal) IgM = 33 mg/dL (low) IgA < 7 mg/dL (undetectable)	duration of COVID-19 symptoms at admission (day) = 7 chest CT-scan = multifocal ground-glass opacities Admission to ICU (day 4) Mechanical ventilation (day 7) ARDS	CVID breast cancer hypothyroidism Sjogren’s syndrome	–	recovered
8	Gamboa E [38]	A co-infection of HIV and COVID-19 case (confirmed by PCR)	59	Male	–	HIV	Undetectable viral load CD4 + cells/µL = 507 the progressive decline of CD4 + and CD8 + during the disease course—went back up to the previous level after disease recovery (726 cells/µL)	chest CT-scan = bilateral ground-glass opacities	HTN ESRD (on renal replacement therapy) HIV (on ART)	–	Recovered (after 2-week hospitalization)
9	Geretti AM [39]	*N* = 47,592 COVID-19 patients *N* = 122 (0.26% with HIV-infection)	56 (49–62)	Male = 80 (66.1%)	–	HIV	Hemoglobin g/dL = 13.0 Anemia = 39 (36.5%) WBC cells/µL = 6.6 Lymphocyte cells/µL = 1000 Lymphopenia (47.2%) Platelet’s count/ µL = 197 Thrombocytopenia (24.8%) PT sec = 13.6 Cr µmol/L = 89 GFR ml/min = 75 ALT U/L = 28 ALT > 40 U/L = (31.5%) Glucose mmol/L = 6.8 Hyperglycemia (20.4%) CRP mg/L = 107	Symptom duration (day) = 5 Current or former Smoker (30.9%)	Previous comorbidity (74.6%) Chronic respiratory disease (10.8%) Asthma (10.3%) Cardiovascular disease (17.1%) dementia (2.5%) Diabetes mellitus, no complications (13.7%) Diabetes mellitus, with complications (7.7%) malignancy (3.4%) Hematological disease (3.4%) Obesity (17%) chronic Renal failure (14.1%) chronic neurological disorder (6.9%) liver disease (7.6%)	HIV infection does not increase admission to critical care HIV infection increases the risk of COVID-19’s mortality In young patients, higher mortality was observed among HIV-infected cases (21.3%) Adjusted hazard ratio = 2.87	26.7%
10	Gervasoni C [33]	*N* = 47 Co-infection HIV and COVID-19	51 (± 11)	Male = 36 (77%),	–	HIV	CD4 + cells/µL = 636 (± 290) HIV viral load < 20 copies/ml = 44 (94%)	Disease duration (day) = 14 COVID-19 diagnosis: laboratory confirmed (60%), clinically suspected (50.8%) Hospital admission: 13 (28%) Pneumonia 12(25%)	Previous comorbidity (64%) Dyslipidemia (31.9%) Hypertension (29.8%) Hepatitis C or B co-infection (10.64%) renal disease (8.5%) Diabetes mellitus (6.4%) epilepsy (2%) cardiovascular disease (4.3%) malignancy (6.4%) gastritis (4.3%) organ transplant (2.1%) Chronic respiratory disease (4.3%)	HIV-infected patients with COVID-19 are at the same risk of severe disease or death as the normal population	4.26%
11	Hadi YB [29]	*N* = 50,167 COVID-19 patients (HIV-infected = 404)	48.2 (SD 14.2)	Male = 285 (71%)	–	HIV	CRP mg/L = 71.15 LDH U/L = 372.45 (SD = 291.05) ESR = 52.89 ALT U/L = 37.2 AST U/L = 48.43 Bilirubin mg/L = 0.84 Ferritin ng/mL = 23,646.94	ICU admission = 27 (6.7%) Current or former Smoker (13.86%)	Hypertension (46.29%) Chronic respiratory disease (25%) Diabetes mellitus (22.03%) Chronic kidney disease (16.58%)	in unmatched analysis: HIV-infected patients showed higher mortality (risk ratio 1.55) and higher inpatient service need (RR = 1.83) After propensity score matching: no difference in mortality rate, but still higher inpatient service needs in HIV-infected patients	4.95%
12	Härter G [34]	*N* = 33 Co-infection HIV and COVID-19	48 (range 26–82 years)	Male = 30 (90.9%)	–	HIV	Median CD4 + T-cell = 670 cells/µL HIV RNA < 50 copies/ml = 30 (93.75%)	Mild symptoms (76%) severe (6%) critical (18%) requiring mechanical ventilation = 2 (6.1%) ICU admission = 6 (18.2%) Hospital admission (42%)	Previous comorbidity (60.0%) Hypertension (30.3%) Chronic respiratory disease (18.2%) diabetes mellitus (12.1%) cardiovascular disease (9.1%) chronic Renal failure (6.1%) Hepatitis B Co-infection history (15.2)	No increased morbidity and mortality among symptomatic COVID-19 co-infection with HIV on ART	9%
13	Ho H-e [30]	*N* = 93 Co-infection HIV and COVID-19	58 (52–65)	Male = 67 (72%)	–	HIV	during COVID-19 illness: significant lymphopenia and decreased CD4 + T-cell counts and percentages Nadir CD4 + , cells/µL = 220 CD4% = 23 HIV RNA < 50 copies/ml (89.1%) Nadir WBC, cells/µL = 5100 Nadir ALC, cells/µL = 900 Nadir ANC, cells/µL = 3500 ALT U/L = 45 AST U/L = 61 Total bilirubin, mg/dL = 0.7 ALP, U/L = 96 Increased levels of inflammatory markers: CRP, mg/L = 137.0 Fibrinogen, mg/dL = 626 D-dimer, μg/mL = 2.6 IL-6, pg/mL = 57.6 IL-8, pg/mL = 42.2 TNF-α, pg/mL = 21.8 IL-1β, pg/mL = 0.3	Hospital admission (77.4%) ICU admission (20.4%) mechanical ventilation = 15 (16.1%) Current or former Smoker (54.8%)	Autoimmune disease (4.3%) Cancer (8.6%) Diabetes mellitus (34.4%) cardiovascular disease (18.3%) Hypertension (52.7%) Chronic respiratory disease (26.9%) chronic kidney disease (17.2%) ESRD (7.5%) organ transplant (5.4%) History of opportunistic infection (24.7%)	Died patients has significant lower nadir absolute lymphocyte count and higher level of Inflammatory markers patients with HIV infection are at risk for severe COVID-19, especially with increased inflammatory markers and immune dysregulation having a worse prognosis	19 out of 93 (20.4%) 19 out of 72 hospitalized individuals (26.4%)
14	Karmen-Tuohy S [27]	*N* = 63 patients hospitalized with COVID-19 (HIV-positive *N* = 21 and matched non-HIV: *N* = 42)	–	–	–	HIV	On admission: WBC = 7200 Hemoglobin = 12.70 Absolute neutrophil count = 5800 Absolute lymphocyte count = 1090 (higher in HIV positives) Ferritin = 679 D-dimer = 333 Troponin = 0.02 Creatine phosphokinase = 239 Procalcitonin = 0.2 Creatinine = 1.14 CRP = 154.5 (higher in HIV positives) LDH = 449.4 absolute CD4 count = 298 CD4 count < 200/mL (31.6%) CD4% = 24 viral load < 50 copies/mL (88.2%)	Length of hospital stay, d = 6 ICU admission = 6 (28.6%) mechanical ventilation = 5 (23.8%) Abnormal initial chest X-ray 19 (90.5%)—(higher in HIV-positive patients)—no difference for abnormal chest X-ray ever-present during this hospitalization Complications: Myocardial infarction = 1 (4.8%) Pulmonary embolism = 1 (4.8%) Deep vein thrombosis = 1 (4.8%) bacterial pneumonia = 3 (14.3%)	–	No difference in length of the hospital stays, rates of ICU admission, mechanical ventilation, and mortality	28.6%
15	N.S.C. van Oers [23]	An infant with X-SCID	Infant	Male	X-SCID	–	–	hepatitis	X-SCID	He received a haploidentical CD34 selected stem cell transplant	0%
16	Douglas Tremblay[40]	24 cancer patients treated with convalescent plasma for severe COVID‐19	69	58.3%male	–	Cancer Hematologic malignancy (58%) Solid malignancy (42%)	Neutropenic (0%) Lymphocytopenic (58.3%)	95.9% required supplemental oxygen 16.7% require NIPPV 12.5% were intubated	HTN 62% DM 33% CKD 29% CAD 20% COPD 20% CHF Obesity 20%	–	41.7%
17	K. Sigel [35]	88 PLWH hospitalized with COVID-19	61	75%male	–	HIV	WBC = 7.2 Creatinine = 1.2 D-dimer = 1.98 CRP = 119 Ferritin = 692 IL6 = 64.1 Prolactin = 0.21 LDH = 428	Moderate/Severe 17% Severe 21%	DM 27% HTN38% Obesity 10% Cirrhosis 6% COPD 9% CAD 7% CKD 22% Organ transplant 5% Cancer 17%	no differences in adverse outcomes associated with HIV infection for hospitalized COVID-19 patients compared with a similar comparison group	21%
18	A. M. Shields [21]	100 patients with PID and symptomatic SID	PID: 30 SID: 15	44%male	*N* = 60 (CVID Undefined primary antibody deficiency XLA)	*N* = 40 (Chronic lung, Cardiovascular, Chronic liver disease, Diabetes mellitus)	–	–	–	Patients with PID and symptomatic SID showed a higher risk of morbidity and mortality from COVID-19	Infection–fatality ratio PID: 20% SID: 33%
19	N. Shalev [36]	31PLWH Hospitalized for Coronavirus	60.7	Male 77%	–	HIV	Lymphocyte = 12.6 CRP = 182 Ferritin = 1356 d-dimer = 6.9 Procalcitonin = 2.2	Low-flow nasal cannula 42% Non-rebreather mask 23% Mechanical ventilation 26% Fever 74% Radiologic changes 65%	HTN67% DM 42% CKD 23% COPD 26% Obesity 33%	Clinical outcomes were comparable to patients who investigated in other hospitalized cohorts	25.8%
20	S. R. Nagarakanti [37]	23 HIV patients admitted for COVID‐19	59	Male 51%	–	HIV	WBC = 6.6 Lymphocytes = 16% HGB = 13 PLT = 310 D-dimer = 193 Albumin = 2.1 Procalcitonin = 3.1 CPK = .025 LDH = 240	required ICU admission 9% HR > 100 52% RR > 20 83% Ambient air 74% Nasal Cannula 9% NRB 13% HFNC 4%	HTN 65% CKD 48% DM 30% CAD 9% COPD 4%	Compare to the matched controls, no difference in mortality and critical care needs for HIV patients	13%
21	H. Miyashita [31]	161 HIV patients with COVID-19	60	–	–	HIV	–	ICU admission 22% Intubation 12%	HTN 46% DM 29% CKD 24% Dyslipidemia 34% HF 9%	Young COVID‐19 patients with HIV infection are at a higher risk for mortality and invasive intubation, compared with non-HIV patients	14%
22	I. Meyts [26]	94 patients with IEI with SARS-CoV-2 infection	30	65%male	Primary antibody deficiency (56%) immune dysregulation syndrome (9.6%) phagocyte defect (6.4%) autoinflammatory disorder (7.4%) combined immunodeficiency (15%) innate immune defect (3%) bone marrow failure (2%)	–	–	ICU admission 20% Asymptomatic 11% Mild 25% Hospitalization 63%	–	Patients with IEI mainly experience a mild form of the disease same risk factors predict severe disease of IEI and normal population	9.5%

Data are represented as median (IQR), mean ± SD, or N (%)

### Primary immunodeficiency disorders (PID)

Delavari et al.’s study [24] showed considerably higher mortality than the general population among PID patients with combined immunodeficiency and immune dysregulation, even with the same rate of COVID-19 infection incidence. In line with Delavari, A.M. Shields's study claimed patients with PID and symptomatic SID are at higher risk of morbidity and mortality from COVID-19[21]. However, the case studies by Fill et al. and van Oers et al. [23, 25] represented cases with PID, which both recovered successfully without a fulminant course of disease. The authors hypothesized that immunosuppression might help prevent severity and mortality due to COVID-19. In addition, a study by B. Cohen et al. [19] suggested that patients with immunodeficiency are not at a greater risk for COVID-19 mortality and disease severity. Besides, Meyts I. et al.’s study [26] on patients with inborn errors of immunity (IEI) confirmed comparable manifestations and risk factors for mortality and morbidity among these patients and the general population.

### Secondary immunodeficiency (SID)

The primary outcome that was sought in the immunodeficient patients in the present review was the mortality rate. The mortality rate ranges from 0.09% [17] to 28.6% [27] in HIV patients, as a major part of secondary immunodeficient. Five studies [17, 28–31] found HIV infection as a deteriorating factor in the severity and outcome of the COVID-19 patients, with the catastrophic outcome especially in the black race [17, 28]. However, eight studies [18, 27, 32–37] suggested that HIV-infected patients have no worse or higher incidence than the normal population.

### Risk of bias/quality assessment

All the studies had acceptable quality based on the NOS; therefore, no studies were excluded from this systematic review due to poor quality. The most encountered problem that could contribute to a possible introduced risk of bias was the lack of matching for the two groups of cases and controls in some studies (Table 3).

**Table 3:** Risk of bias assessment of the studies based on the Newcastle–Ottawa scale (NOS)

Study	Selection (Out of 4)	Comparability (Out of 2)	Exposure/outcome (Out of 3)	Total score (Out of 9)
Bhaskaran K [17]	4	2	3	9
Cabello A [18]	4	2	3	9
Childs K [28]	3	–	3	6
Cohen B[19]	4	–	3	7
Del Amo J [32]	4	–	3	7
Delavari S [24]	4	–	3	7
Fill L [25]	3	–	3	6
Gamboa E [38]	3	–	3	6
Geretti AM [39]	4	2	3	9
Gervasoni C [33]	4	–	3	7
Hadi YB [29]	4	2	3	9
Härter G [34]	4	–	3	7
Ho H-e [30]	4	2	3	9
Karmen-Tuohy S [27]	4	2	3	9
N.S.C. van Oers [23]	3	–	3	6
Douglas Tremblay [40]	4	–	3	7
K. Sigel [35]	4	2	3	9
A. M. Shields [21]	4	1	3	8
N. Shalev [36]	4	–	3	7
S. R. Nagarakanti [37]	4	2	3	9
H. Miyashita [31]	4	–	3	7
I. Meyts [26]	3	–	3	6

## Discussion

Due to emergence of COVID-19 pandemic, understanding the difference between the reaction of the immune system in patients with and without underlying conditions has been a matter of debate [41, 42]. This systematic review analyzed a total of 22 different studies that met the inclusion criteria and sought to establish the predictors and mortality rate in COVID-19 patients who have weak immune systems otherwise known as immune-compromised or immunodeficient patients. The different immune system conditions as seen in suppressive situations have been attributed to the severity, mortality, and high infection rate of the diseases [15]. This category of patients has been a point of attention from the start of the COVID-19 pandemic, since they are more prone to infectious diseases and classified as one of the high-risk groups of patients. Breakdown of the study type included in our analysis can be seen in Table 2 and the flow of inclusion criteria is represented in Fig. 1. Immunodeficient patients are widely categorized into primary and secondary immunodeficiencies with markedly decrease or absent immune cells or system function, and thus, for a better assortment of this study, we separated the cases into these two categories of patients.

The first one is the cases reported with the PID, such as CVID, humoral immunodeficiency, etc. PID refers to a diverse set of illnesses caused by flaws in the immune system's development and/or function. PIDs have a wide range of signs and symptoms, but the majority of them involve an elevated susceptibility to infection, with several of them resulting in substantial disease-related adverse outcomes (morbidity and mortality). This point of view might seem logical and reasonable in the first place and lead us to the idea that having PID might increase the mortality of the COVID-19 patients as stipulated by the findings of Delavari et al. study in which they found that even though COVID-19 is normally a minor disease in children and adolescents (due to low ACE2 receptor expression and effective adaptive immunity), a small percentage of individuals, particularly PID patients, may suffer severe disease necessitating transfer to an intensive care unit (ICU) and even death[24]. Meyts I et al. also found that younger patients with inborn errors of immunity (IEI) were more severely affected and more frequently admitted to ICU compared with the general population. These findings warrant a recommendation for further stringent personal protective measures for patients affected by IEI [26]. However, this hypothesis might not seem that logical if we change our point of view. One of the main causes of the COVID-19 symptoms is the patient’s immune system reaction leading to a cytokine storm in severe cases. From this point of view, a suppressed immune system might lower the chance of appearance of these symptoms. Referring to Fill et al. findings which state that the immunosuppression may be beneficial in preventing mortality, symptoms of hyperinflammation, and cytokine storm brought about by the SARS-CoV-2 virus interaction with the host immune responses, including interleukin (IL)-2, IL-6, IL-7, ferritin, and tumor necrosis factor-alpha among others[25]. In another study by van Oers et al. the lack of T cells, NK cells, and functional B cells in their patient did not lead to a fulminant or severe respiratory compromise, which is typical of COVID-19 disease [23].

Further analysis of our study results indicates that COVID-19 patients with primary immunodeficiencies (PIDs) have a markedly high mortality rate as well as a higher risk of infection with the SARS-CoV-2 virus. This may be highly attributed to the absence or poor function in one or more components of the immune system. It seems to be in line with a multi-center international analysis of 94 patients that were identified with an inborn error of immunity, of which 53 (56%) had primary antibody deficiency. They reported more than a third of all patients had CVID, but four of them died (45% of all deaths) [26]. Immunocompromised patients may be more susceptible to direct viral insult, which can cause organ damage and lead to morbidity and mortality, but those with combined immunodeficiency are at a larger risk [43]. In contrast to this, our study found that COVID-19 patients with CVID do not present additional mortality rates. Similarly, despite the presence of comorbidities, COVID-19 individuals with CVID had a minimal illness and did not require hospitalization. As a result, certain adaptive immune components do not appear to be required in management of SARS-CoV-2 infection. Rather, by lowering immunological-mediated consequences, these adaptive immune deficits may contribute to a milder course [26]. This, however, suggests that COVID-19 patients with CVID, compared with other forms of PIDs and the normal population, may be at a higher risk of infection but with a lower mortality rate, indicating a good recovery rate for this category of patients [19]. However, PID patients under the category of SCID (Severe Combined Immunodeficiency) had a seemingly high risk of infection and death in our study. In concordance with our study, COVID-19 infection was found to be the deadliest among PID individuals in patients with SCID and familial hemophagocytic lymphohistiocytosis (FHL). This idea backs up the prior theory that SARS-CoV-2 is more deadly in patients with cellular immunodeficiency and immunological dysregulation, with a nearly 150-fold increased risk of death [24]. Another factor that appeared to influence the mortality and infection rate has been attributed to PID patients with underlying diseases before infection with COVID-19. Factors such as lymphoproliferation and history of lower respiratory infection in PID patients were observed in this group of people, recording high mortality rates.

SID is an acquired immunodeficiency due to a disease or environmental cause, such as malnutrition, human immunodeficiency virus (HIV), or medical treatment (e.g., radiotherapy, chemotherapy). Our analysis presented ten studies reporting a mortality rate > 10% and seven studies presented a mortality rate of < 10% in secondary immunodeficient subjects with COVID-19. We settled on case fatality > 10% to be high. In all SID–COVID-19 cases, mortality ranged from 0% to as high as 41.7% [19, 44]. The most prominent SID among included studies was HIV and lesser cases involved cancer, hematologic malignancy, solid malignancy, etc. Some studies showed increased rates of mortality and morbidity and some showed the same or less rates than normal people. Bhaskaran K et al. discovered that individuals infected with HIV in England have an increased risk of COVID-19 death accounting for demographic and lifestyle-related factors, supporting the first point of view in the second type of immunodeficiency illnesses [17]. Comparable to them, Hadi YB et al. discovered that COVID-19 crude mortality is greater in HIV-positive individuals than in non-HIV patients; nonetheless, propensity-matched studies indicated no differences in outcomes, indicating that perhaps the higher mortality is attributable to the HIV patients' higher load of underlying conditions and risk factors for severe COVID-19 [29].

The HIV attacks and impairs the body’s host defense system and causes immune deficiency. This immune deficiency lowers the severity of the immune system reactions against the SARS-CoV-2 virus, such as cytokine storms and the side effects that are caused by these reactions. It is important to note that if there are no comorbidities associated with HIV, the cytokine storm will be inhibited, and patients will have less serious symptoms and consequently low mortality rates [31]. However, according to our study, the case fatality rate is seemingly high in HIV patients with COVID-19, suggesting a higher death risk for this group of patients. Nevertheless, this high mortality rate in HIV–COVID-19 patients could be attributed to comorbid conditions and underlying diseases in this category of patients in our study. This strongly supports the hypothesis that HIV–COVID-19 patients have less serious symptoms and a low mortality rate due to the suppression of cytokine storm and its related effects provided that there are no comorbidities. The underlying diseases that could contribute to the high death rate include hypertension (HTN), diabetes mellitus (DM), end-stage renal disease (ESRD) or chronic kidney disease (CKD), cardiovascular disease (CD), hepatitis B and C, dyslipidemia, neurological disease (mainly dementia), rheumatoid arthritis (RA), chronic obstructive pulmonary disease (COPD), congestive heart failure (CHF), and coronary artery disease (CAD) [32]. Other demographic factors such as obesity and age > 70 years play an important role in the death rate for this group of patients. Further analysis indicated that SID cases of cancer, hematologic malignancies, and solid malignancies presented the highest case mortality rate > 40% in our study. This firmly suggests that COVID-19 patients in this category have an increased risk of infection and expiration. Interestingly, there was an intersection in underlying diseases that may have influenced the mortality rate in both PID–COVID-19 patients and SID–COVID-19 patients. Though HTN and DM intersected in both groups, no mortality was recorded in the PID cases, hence cannot be implicated in the death rate in this group. The reverse was observed in the SID cases [33, 45].

On the contrary, we have some studies in which the findings are controversial. These studies have the second point of view in the second type of immunodeficiency. Cabello A et al. findings show that neither the HIV severity nor the type of ARV treatment seems to influence the outcome of COVID-19 [18]. Similar to this point of view, Gervasoni C et al. found that patients infected with HIV and SARS-CoV-2 are not at a higher risk of developing severe disease or death than patients without HIV [33]. Similar to the previously mentioned studies, a study by Karmen-Tuohy S et al. shows that HIV coinfection did not affect the manifestation, hospital course, or prognosis of SARS-CoV-2 patients in comparison with matched non-HIV patients [27].

## Limitations and recommendations

Clinical management and therapy of these two groups were not considered collectively, since the studies included had different clinical management of the disease and others did not include the type of therapy used. It is important to note that the therapy involved in each case could influence the clinical outcome (recovery or risk of expiration), since most COVID-19 therapies are trials and patient management. Therefore, further studies are needed and highly recommended to focus on this aspect for patients suffering from both immunodeficiencies and COVID-19. This will enable clinicians to administer precise management and subsequently reducing COVID-19 mortality rates in immunodeficient patients.

## Conclusions

The review of recent studies represents two points of view in both categories of immunodeficiency diseases. The classical viewpoint stipulates that all immunodeficient patients are at a higher risk of infection and consequently leading to a poor adverse outcome of COVID-19 serve as our first school of thought. However, our second perspective is in sharp contrast to this idea in which immunodeficiency may play a less important role in raising the rate of death by lowering the severity of the cytokine storm and its consequences. Both hypotheses have a noticeable number of supportive studies. Nonetheless, it is critical to recognize the role of comorbidities, such as COPD, DM, hypertension, CKD, history of lower respiratory infection, CAD, etc., and demographic factors, such as obesity and age > 70 years, in causing high COVID-19 mortality rates especially in SID individuals.

## Data Availability

The authors stated that all information provided in this article could be share.
